# One-Year Changes in Depressive Symptoms and Cognitive Function Among Brazilian Older Adults Attending Primary Care

**DOI:** 10.3390/healthcare13070807

**Published:** 2025-04-03

**Authors:** Fernanda Maria Silva Rivoli, Antonio Pedro Gabriel Monteiro Galhardo, Giancarlo Lucchetti, Lízia Abreu Esper, Yan Lyncon Ribeiro, Gerson de Souza Santos, Helena José, Luís Sousa, Gail Low, Luciano Magalhães Vitorino

**Affiliations:** 1 Faculty of Medicine of Itajubá, Itajubá 37502-138, MG, Brazil; ferivoli@gmail.com (F.M.S.R.); antoniopedrofmit@gmail.com (A.P.G.M.G.); liziaaesper@hotmail.com (L.A.E.); yan.obras2018@gmail.com (Y.L.R.); 2 School of Medicine, Federal University of Juiz de Fora (UFJF), Juiz de Fora 36038-330, MG, Brazil; g.lucchetti@yahoo.com.br; 3 Department of Medicine, Centro Universitário Ages, Paripiranga 48430-000, BA, Brazil; gerson.s.santos@ages.edu.br; 4 Atlântica School of Health, 2730-036 Barcarena, Portugal; hjose@uatlantica.pt (H.J.); luismmsousa@gmail.com (L.S.); 5 Health Sciences Research Unit: Nursing (UICISA: E), Nursing School of Coimbra, 3004-011 Coimbra, Portugal; 6 RISE-Health, Faculty of Medicine, University of Porto, 4200-319 Porto, Portugal; 7 Comprehensive Health Research Centre, University of Évora, 7000-801 Évora, Portugal; 8 Faculty of Nursing, MacEwan University, Edmonton, AB T5J 4S2, Canada; lowg4@macewan.ca

**Keywords:** aging, depression, cognitive dysfunction, geriatric psychiatry, longitudinal studies

## Abstract

**Background**: Aging is a global phenomenon closely associated with changes in cognitive function and mental health. These conditions substantially burden public health systems and adversely affect the quality of life of older adults. This study aimed to examine changes in depressive symptoms and cognitive function over a 12-month follow-up period in a cohort of Brazilian older adults attending primary care. **Methods**: This observational longitudinal study included a randomized sample of individuals aged ≥60 years residing in São Paulo, Brazil, and registered at a Primary Healthcare Unit (PHU). Data collection involved administering a sociodemographic and health questionnaire along with two validated instruments: the Geriatric Depression Scale-15 (GDS-15) and the Mini-Mental State Examination (MMSE). Linear regression models were used for the analyses. **Results**: A total of 368 older adults were included, with 63% being men and a mean age of 74.65 years. After one year, depressive symptoms showed a notable increase, with the mean GDS-15 score rising from 5.97 to 7.48 (Cohen-d = 0.542). Likewise, there was a decrease in the mean MMSE score ranging from 19.11 to 18.88 (Cohen-d = 0.216). Adjusted regression analyses revealed that depressive symptoms at baseline (B = 0.696; *p* = 0.048; R^2^ = 0.19) and cognitive function at baseline (B = 0.444; *p* < 0.001; R^2^ = 0.26) were predictive of their respective deteriorations over the follow-up period. **Conclusions**: Depressive symptoms and cognitive decline place a significant burden on public health systems in aging societies. These findings underscore the importance of continuous monitoring and early intervention strategies to mitigate their impact and enhance the quality of life for older adults.

## 1. Introduction

The global demographic shift toward population aging carries substantial implications for public health systems worldwide [1]. In Brazil, approximately 14.7% of the population is aged 60 years or older. This proportion is projected to reach 64 million individuals by 2050 [1,2]. In comparison, 16.8% of the U.S. population and 28.4% of Japan’s population are already aged 65 or older [2]. These demographic dynamics pose complex challenges to healthcare systems, especially regarding the management of chronic conditions, geriatric syndromes, and neuropsychiatric disorders such as late-life depression and cognitive impairment. Such conditions markedly impair functional independence and contribute to escalating healthcare expenditures [3,4].

Depression is among the most prevalent mental health conditions affecting older adults, with a global prevalence of 28.4%. Its frequency varies across regions, influenced by cultural and socioeconomic factors that shape the onset and progression of symptoms [5,6]. Similarly, cognitive decline represents another major concern, impacting 21.2% of older adults in long-term care facilities globally [6].

Evidence suggests both conditions frequently co-occur, reinforcing each other in a cycle that exacerbates both mental and cognitive health. Proposed underlying mechanisms include neuroinflammation, dysregulation of the hypothalamic–pituitary–adrenal (HPA) axis, and cerebrovascular dysfunction, which may jointly contribute to this bidirectional relationship [7]. Studies conducted in high-income countries indicate that depressive symptoms and cognitive decline become markedly more prevalent after the age of 75 [7,8].

In Brazil, depressive symptoms and cognitive decline present growing public health challenges, with national data indicating a rising prevalence, particularly among low-income and underserved populations [9,10]. Depressive disorders have become significant contributors to disability-adjusted life years (DALYs) in the country, with rates steadily increasing since 1990 [10].

Despite this, there remains a notable scarcity of longitudinal studies examining the trajectory of depressive symptoms and cognitive function in Brazil. Existing research is predominantly cross-sectional and often overlooks cultural and socioeconomic factors. Additionally, studies focusing on older adults utilizing primary healthcare services—an integral component of Brazil’s healthcare system—are limited. Primary care settings are particularly relevant due to their accessibility, high patient volume, and potential for early identification and intervention. The scarcity of longitudinal studies hinders a comprehensive understanding of the relationship between depressive symptoms and cognitive decline in older adults. Although such studies allow for the observation of temporal changes, the specific influence of clinical and demographic factors on the progression of these conditions often remains underexplored. Evidence generated through longitudinal designs can still support the development of targeted community-based screening strategies, interventions, and policies [11].

Addressing these gaps is vital for improving both clinical practice and healthcare delivery. Depressive symptoms and cognitive decline significantly impair older adults’ functionality and increase their reliance on healthcare services [12]. These conditions are often underdiagnosed in primary care, resulting in delayed treatment and missed opportunities for prevention [13].

To address these issues, this study aims to examine changes in depressive symptoms and cognitive function over a 12-month follow-up period in a cohort of Brazilian older adults attending primary care. Understanding their natural course may inform timely identification and early, targeted intervention among at-risk older adults [13]. The findings from this study may inform broader healthcare strategies by supporting the development of community-based interventions and guiding policy initiatives aimed at promoting healthy aging.

## 2. Materials and Methods

### 2.1. Study Design

This was an observational, longitudinal study (12-month follow-up), conducted among older adults (≥60 years) residing in São Paulo, Brazil. The study was conducted between 2018 and 2019 and was approved by the Research Ethics Committee of the São Paulo Municipal Health Department (approval number 2.961.352). All participants provided written informed consent before data collection at both baseline and follow-up. The study adhered to the ethical principles outlined in the Declaration of Helsinki.

### 2.2. Participants and Eligibility Criteria

A total of 400 older adults (aged ≥60 years) registered at the Marcus Wolosker Belenzinho Primary Healthcare Unit (PHU) in São Paulo were recruited at baseline. This PHU is part of the Unified Health System (Sistema Único de Saúde, SUS), which serves as the primary point of entry for healthcare services. The PHU is situated within an area that, in 2019, was home to some 40,000 persons, among whom 11% were aged 60 years or older. The SUS aims to ensure that primary healthcare facilities like this one address around 80% of the healthcare needs of older persons nationwide, as per the guidelines of Brazil’s Ministry of Health.

### 2.3. Sample Size

Sample size calculations were performed using G*Power 3.1 software to ensure sufficient power for multivariate regression analyses. Assuming a small effect size (f^2^ = 0.10), α = 0.05, power (1 − β) = 0.99, and 7 predictors, a minimum sample size of 300 participants was required. To account for potential losses during follow-up, the sample size was increased by 25% [14], resulting in the recruitment of 400 participants. This final sample size is sufficient to perform robust statistical analyses should there be attrition.

### 2.4. Procedures at Baseline and Follow-Up

At baseline (Time 0), participants were randomly selected from PHU registries using patient identification numbers. Simple random sampling was applied using a computer-generated process in Microsoft Excel, in which patient record numbers were randomly drawn to compose the sample. Data collection was conducted between March and July 2018 during scheduled nursing consultations by a trained nurse with over 10 years of primary healthcare experience. The interviews lasted approximately 40 min and were conducted in private settings to ensure confidentiality. At this stage, participants were informed about the 12-month follow-up and received a scheduled return date. The same nurse who conducted the baseline assessments coordinated follow-up by reinforcing return appointments during routine care, which helped ensure participant retention.

At follow-up (Time 1), assessments were conducted between February and August 2019 using the same protocol. Participants were contacted via phone and scheduled for interviews at the PHU. The follow-up was completed with 368 participants, and all data were collected by the same experienced nurse to maintain consistency.

### 2.5. Measures

#### 2.5.1. Independent Variables

Sociodemographic and health data, including age (years) and gender (male or female), were collected. So too were health-related variables: daily medication use (yes or no), daily number of medications, and the presence of chronic diseases (“Do you have any chronic conditions or diseases?” yes or no). Chronic conditions were defined as those lasting three months or longer, with examples provided, such as hypertension, diabetes, depression, heart disease, arthritis, and cancer. Additional variables included polypharmacy (use of five or more medications daily, yes or no) and a history of falls in the last 12 months (yes or no).

#### 2.5.2. Dependent Variables

Depressive symptoms were assessed using the Geriatric Depression Scale-15 (GDS-15), a 15-item instrument validated in Brazilian Portuguese [15]. Scores range from 0 to 15, with higher scores indicating more severe depressive symptoms. The scale includes items such as “Do you feel that your life is empty?” with response options of Yes (1 point) or No (0 points), and “Do you feel happy most of the time?” with responses of Yes (0 points) or No (1 point). A total score of 6 or more suggests the presence of depressive symptoms. In the present sample, the GDS-15 demonstrated good internal consistency, with a Cronbach’s alpha of 0.83.

The Mini-Mental State Examination (MMSE), a widely used tool for cognitive function screening in older adults, has been validated in Brazilian Portuguese [16]. Scores range from 0 to 30, with higher scores indicating better cognitive function. Items include “What is today’s date?” under the domain of orientation to time, with responses scored as Correct (1 point) or Incorrect (0 points), and “Spell the word ‘WORLD’ backward”, under attention and calculation, with scoring based on full correctness (1 point) or any errors (0 points). The MMSE assesses various domains, including memory, language, and visuospatial skills, with adjustments for educational differences in Brazilian populations [17,18]. In this study, MMSE cutoff scores followed the Brazilian context: 13 for illiterate individuals, 18 for those with up to 8 years of schooling, and 26 for individuals with more than 8 years of education [18]. For the MMSE, internal consistency analysis in the current sample yielded a Cronbach’s alpha of 0.68, indicating an acceptable level of reliability.

### 2.6. Statistical Analysis

All statistical analyses were performed using SPSS version 26. Cases with missing data were excluded using complete-case analysis. No imputation methods were applied, as missingness was minimal and randomly distributed. Descriptive statistics were performed for continuous and categorical variables. Paired *t*-tests were used for within-group comparisons of continuous variables, and McNemar’s test was applied for categorical variables. Effect sizes were calculated using Cohen’s d, with thresholds for small (d ≤ 0.2), medium (d ≈ 0.5), and large (d ≥ 0.8) effects [18]. To examine associations between depressive symptoms (GDS-15) and cognitive function (MMSE), simple and multiple linear regressions were used. Multivariate linear regression models adjusted for clinically relevant covariates (*p* < 0.05) included the number of medications, self-rated health, polypharmacy, and a history of falls. All analyses were performed with a significance level of *p* < 0.05 and a 95% confidence interval (CI).

## 3. Results

Of the 400 older adults recruited at baseline, 368 (92.0%) completed the 12-month follow-up. The majority were women (n = 293; 73.1%), with a mean age of 72.5 years (SD = 7.3). Most participants had low educational attainment, with 242 (60.6%) having ≤8 years of schooling and 89 (22.3%) reporting no formal education. Regarding marital status, 195 (48.9%) were married or cohabiting. A total of 253 participants (63.3%) reported monthly family income of up to twice the minimum wage. At baseline, 148 (36.9%) met the criteria for polypharmacy, 208 (51.9%) had multimorbidity (≥2 chronic conditions), and 139 (34.8%) reported at least one fall in the previous year. These characteristics are detailed in Table 1.

Table 2 shows that scores on the Geriatric Depression Scale (GDS-15) increased significantly from 5.97 (SD = 2.91) to 7.48 (SD = 2.65) (*p* < 0.001), yielding a moderate effect size (d = 0.542). Concurrently, there was a slight (d = 0.216) but significant decline in Mini-Mental State Examination (MMSE) scores, from 19.88 (SD = 2.92) to 19.11 (SD = 2.97) (*p* < 0.001). Cognitive decline, while modest, was statistically significant.

In Table 3, the adjusted linear regression analysis revealed that higher levels of depressive symptoms at baseline were significantly associated with higher depressive symptom levels at 12 months (B = 0.696; *p* = 0.048). Similarly, lower cognitive function at baseline (Time 0) predicted greater cognitive decline one year later at Time 1 (B = 0.444; *p* < 0.001).

## 4. Discussion

This longitudinal study provides important insights into the progression of depressive symptoms and cognitive decline among older adults utilizing primary healthcare services in Brazil. Over a 12-month period, we observed a significant worsening of depressive symptoms, alongside a modest but statistically significant decline in cognitive function.

The observed increase in depressive symptoms, as measured by the GDS-15, aligns with prior studies highlighting a growing burden of emotional distress among older adults over time [19,20,21]. However, in our sample, we observed a moderate statistically significant increase in mean GDS-15 scores after one year.

We also found substantial increases in polypharmacy (from 36.95% to 67.12%), a factor known to exacerbate depressive symptoms [22,23]. Polypharmacy is commonly defined as the concurrent use of five or more medications, whereas excessive polypharmacy refers to the use of ten or more medications [24,25]. In older adults, polypharmacy has been associated with a significantly higher risk of depression, largely due to adverse drug interactions, side effects, and the cognitive burden of complex medication regimens [22].

Polypharmacy can be associated with multiple adversities, including dementia, enhanced symptoms of depression, and even death [26]. Robust evidence indicates that managing complex medication regimens may reduce social motivation and contribute to anxiety, particularly in later life [27,28,29]. We examined data over one year of time. Longer-term observations are warranted to further uncouple links between medication regimens and depressive symptoms.

Cognitive decline, assessed through the MMSE, was modest but statistically significant, corroborating findings from other longitudinal studies involving community-dwelling older adults [30,31]. Despite a small effect size, the steady decline in MMSE scores highlights the vulnerability of cognitive function in aging populations, especially in individuals with multimorbidity and polypharmacy [24,32,33,34,35,36].

Notably, the rise in polypharmacy and a history of falls likely further exacerbated cognitive decline [32,37,38]. Cognitive impairment is known to increase fall risks over time [33,39]. Polypharmacy is a double-edged sword with many ugly sides. Other pernicious companions include a slower gait [40], frequent hospital admissions, and non-beneficial medication prescribing [33]. A slower gait has enhanced some community-dwelling older persons’ risk of falling for up to one year [41]. Less capacity to get out and about of one’s own volition, albeit from falls or other traumas, and shrinking social circles can aggravate emotional distress in later life [28,29]. Some older people experiencing physically traumatic events report taking 10 or more medications a day [36]. Persistent complex regimens have put others at a significantly higher risk for fall-related injuries in both hospital and outpatient settings [25].

These findings reinforce the need for regular reviews of pharmacological regimens, focusing on therapeutic necessity and minimizing exposure to fall-risk-increasing drugs (FRIDs) such as narcotics, diuretics, beta-blockers, and antipsychotics [25,34,39,42,43]. Cognitive impairment can complicate clinical assessment by masking or mimicking symptoms of other conditions, especially in individuals living with dementia [32]. Therefore, comprehensive evaluation and cautious deprescribing strategies are essential in this population.

Best practices for reducing opioids, benzodiazepines, and other FRIDs should include fall and depression-related risk education and transparent co-designed follow-up plans for patients and their families. Patients without family would benefit from a PHU advocate such as a Nurse Practitioner. Good deprescribing duly considers older persons’ overall health and comfort [34,44].

Ultimately, older persons are the ones who have to live with the psychological impacts of medication regimens, day in and day out. Older persons residing in 20 different countries have reported that quality living is about being one’s own health steward, both with clarity of mind and with healthcare practitioners knowing what they value and expect, and their immediate living environment [45]. This cannot be over-emphasized. In this study, as others point out [46], depressive symptoms and cognitive decline can and do co-exist in later life. Depression may impair cognitive processes, such as memory, attention, and executive function, while cognitive decline can exacerbate depressive symptoms by reducing independence and quality of life [47].

While this bidirectional relationship is well-recognized, the temporal sequence remains unclear. A meta-analysis of community-based cohort studies found that late-life depression nearly doubles the risk of developing dementia [48]. Additionally, psychosocial stressors—including perceived stress, social isolation, and low emotional support—have been independently associated with accelerated cognitive decline in older adults [49]. These findings reinforce the hypothesis of shared neurobiological mechanisms and highlight the need for integrated assessment and management strategies in primary care.

We implore healthcare practitioners to keep this vicious circle in mind early, at the forefront of co-decision-making and co-design of follow-up planning.

Another best practice is establishing a community of care wherein patients and practitioners steer the clinical decision-making rudders [50]. Shared decision-making and clarity of mind are ideal steadfast companions, while practitioner-devised complex drug regimens and lived cognitive impairment are not. Older people have long argued that having a stake in decision making is important [51]. This can entail exploring, early on, older persons’ preferences for the number of medications and their perceived impacts and capacity to manage them.

These findings have important implications for clinical practice. The observed increase in depressive symptoms over 12 months underscores the need for the early identification and timely treatment of depression in older adults. Routine depression screening in primary care settings using validated tools like the GDS-15 should be a priority early on to enhance diagnostic accuracy [52]. Early interventions, including both pharmacological and non-pharmacological approaches such as cognitive-behavioral therapy and social engagement programs, are also recommended to keep depressive symptoms in tow. Primary care providers should incorporate tools like the MMSE or Mini-Cog into routine evaluations, particularly for patients with known risk factors such as polypharmacy and a history of falls [53,54].

Finally, the study highlights the urgent need for a multidisciplinary approach in primary care. Given the interconnected nature of depressive symptoms and cognitive decline, collaboration among geriatricians, psychologists, and primary care providers is essential. Comprehensive care models addressing both mental and physical health could improve outcomes for older adults while reducing long-term healthcare burdens [53]. Other essential practitioners helping older persons to co-design follow-up plans include pharmacists, nurse practitioners, and occupation therapists.

Despite its strengths, this study has several limitations. The sample was drawn from a single PHU in São Paulo, which may limit the generalizability of what we found. Additionally, while validated instruments (GDS-15 and MMSE) were used, the reliance on self-reported data for variables such as medication use and fall history may introduce recall bias. Although comorbidities and socioeconomic status were included as covariates in the regression models, their individual contributions to the outcomes were not examined in depth, representing a limitation of this study. Social determinants such as low income, limited access to health services, and reduced social support are known to negatively impact both mental health and cognitive trajectories in aging populations.

Integrating these contextual factors into prevention strategies is essential to reduce disparities and promote healthy aging in primary care settings. Although the study has important strengths, it did not account for several potentially relevant contextual factors, which may have influenced the outcomes. Future research should consider integrating objective measures and examining the influence of key social determinants—such as socioeconomic status, living environment, and access to adjunct therapies and programs—on the progression of depressive symptoms and cognitive decline. Expanding longitudinal studies to diverse settings would also enhance our understanding of these conditions and their broader implications.

## 5. Conclusions

This study demonstrates that depressive symptoms and cognitive decline are significant and interrelated challenges for older adults in primary care settings. The observed worsening of depressive symptoms and modest cognitive decline over 12 months underscores the importance of early detection and intervention.

Our findings advocate for routine screening and the early adoption of comprehensive and co-designed interventions to mitigate the impact of these conditions. Future research should focus on elucidating the mechanisms linking depressive symptoms and cognitive decline and evaluating the effectiveness of co-designated early intervention and follow-up plans. We can best uncouple these links within multi-disciplinary teams with older people helping to steer clinical decision-making rudders.

## Figures and Tables

**Table 1:** Sociodemographic and health profiles of participants at baseline (T0) and 12-month follow-up (T1) ^a^.

Variables	2018 (n = 368)	2019 (n = 368)	*p*-Value
Age (M; SD)	74.65 (7.99)	75.36 (7.79)	0.058
Number of Medications	3.83 (2.02)	4.68 (1.65)	<0.001
	n (%)	n (%)	
Gender			
Male	232 (63.0)	233 (63.3)	>0.999
Female	136 (37.0)	135 (36.7)	
Self-Perceived Health			
Excellent	28 (7.60)	16 (4.35)	
Good	114 (30.97)	97 (26.38)	0.001
Fair	148 (40.22)	137 (37.23)	
Poor	78 (21.21)	118 (32.03)	
Chronic disease			
Yes	338 (91.84)	367 (99.72)	<0.001
No	30 (8.15)	1 (0.27)	
Polypharmacy (≥5 medications)			
Yes	136 (36.95)	247 (67.12)	<0.001
No	232 (63.05)	121 (32.88)	
History of Falls ^b^			
Yes	251 (68.20)	367 (99.80)	<0.001
No	148 (31.80)	1 (0.20)	

^a^ Older adults (n = 32) who participated at the baseline but not follow-up were excluded from this analysis. ^b^ in the 1st 12 months. SD: Standard Deviation.

**Table 2:** Comparison of depressive symptoms and cognitive function of participants (n = 368).

Variables	T0 (n = 368)	T1 (n = 368)	*p*-Value	Effect Size
Depressive Symptoms	Mean (SD)	Mean (SD)		
GDS-15	5.97 (2.91)	7.48 (2.65)	<0.001	0.542
Cognitive Function				
MMSE	19.88 (2.92)	19.11 (2.97)	<0.001	0.216

SD: Standard Deviation; GDS-15: Geriatric Depression Scale-15; MMSE: Mini-Mental State Examination.

**Table 3:** Adjusted linear regression analyses of depressive symptoms and cognitive function at baseline (Time 0) and 12-month follow-up (Time 1).

Variables	B (SE)	Beta	*p*-Value	Adjusted R^2^
GDS-15 *	0.123 (0.047)	0.135	<0.001	0.13
GDS-15 ** (Sociodemographic and Clinical)	0.696 (0.386)	0.110	0.048	0.19
MMSE *	0.465 (0.047)	0.457	<0.001	0.29
MMSE ** (Sociodemographic and Clinical)	0.444 (0.049)	0.437	<0.001	0.26

* GDS-15 and MMSE without adjustment; ** GDS-15 and MMSE adjusted for clinical variables (number of medications, self-perceived health, polypharmacy, history of falls). SE: Standard Error.

## Data Availability

Data will be available upon request (lucianoenf@yahoo.com.br).

## References

[1] Dall T.M., Gallo P.D., Chakrabarti R., West T., Semilla A.P., Storm M.V. (2013). An Aging Population and Growing Disease Burden Will Require a Large and Specialized Health Care Workforce by 2025. Health Aff..

[2] Tramujas Vasconcellos Neumann L., Albert S.M. (2018). Aging in Brazil. Gerontologist.

[3] Prince M.J., Wu F., Guo Y., Gutierrez Robledo L.M., O’Donnell M., Sullivan R., Yusuf S. (2015). The Burden of Disease in Older People and Implications for Health Policy and Practice. Lancet.

[4] GBD 2019 Dementia Forecasting Collaborators (2022). Estimation of the Global Prevalence of Dementia in 2019 and Forecasted Prevalence in 2050: An Analysis for the Global Burden of Disease Study 2019. Lancet Public Health.

[5] Hu T., Zhao X., Wu M., Li Z., Luo L., Yang C., Yang F. (2022). Prevalence of Depression in Older Adults: A Systematic Review and Meta-Analysis. Psychiatry Res..

[6] Chen P., Cai H., Bai W., Zhang Q., Su Z., Tang Y.-L., Ungvari G.S., Ng C.H., Xiang Y.-T. (2023). Global Prevalence of Mild Cognitive Impairment among Older Adults Living in Nursing Homes: A Meta-Analysis and Systematic Review of Epidemiological Surveys. Transl. Psychiatry.

[7] Weyerer S., Eifflaender-Gorfer S., Wiese B., Luppa M., Pentzek M., Bickel H., Bachmann C., Scherer M., Maier W., Riedel-Heller S.G. (2013). Incidence and Predictors of Depression in Non-Demented Primary Care Attenders Aged 75 Years and Older: Results from a 3-Year Follow-up Study. Age Ageing.

[8] Borelli W.V., Zimmer E.R., Bieger A., Coelho B., Pascoal T.A., Chaves M.L.F., Amariglio R., Castilhos R.M. (2022). Subjective Cognitive Decline in Brazil: Prevalence and Association with Dementia Modifiable Risk Factors in a Population-Based Study. Alzheimer’s Dement. Diagn. Assess. Dis. Monit..

[9] Zenebe Y., Akele B., W/Selassie M., Necho M. (2021). Prevalence and Determinants of Depression among Old Age: A Systematic Review and Meta-Analysis. Ann. Gen. Psychiatry.

[10] Bonadiman C.S.C., Malta D.C., de Azeredo Passos V.M., Naghavi M., Melo A.P.S. (2020). Depressive Disorders in Brazil: Results from the Global Burden of Disease Study 2017. Popul. Health Metr..

[11] da Nóbrega I.R.A.P., Leal M.C.C., Marques A.P.d.O., Vieira J.d.C.M. (2015). Fatores Associados à Depressão Em Idosos Institucionalizados: Revisão Integrativa. Saúde Debate.

[12] García-Peña C., Espinel-Bermúdez C., Tella-Vega P., Pérez-Zepeda M.U., Gutiérrez-Robledo L.M., García-Peña C., Gutiérrez-Robledo L.M., Pérez-Zepeda M.U. (2018). Longitudinal Studies and Older Adults Cohorts. Aging Research—Methodological Issues.

[13] Pereira M.E.A., Santos G.d.S., Almeida C.R.d., Nunes K.C.S., Silva M.C.M.d., José H., Sousa L., Vitorino L.M. (2024). Association between Falls, Fear of Falling and Depressive Symptoms in Community-Dwelling Older Adults. Healthcare.

[14] Fewtrell M.S., Kennedy K., Singhal A., Martin R.M., Ness A., Hadders-Algra M., Koletzko B., Lucas A. (2008). How much loss to follow-up is acceptable in long-term randomised trials and prospective studies?. Arch. Dis. Child..

[15] Paradela E.M.P., Lourenço R.A., Veras R.P. (2005). Validação Da Escala de Depressão Geriátrica Em Um Ambulatório Geral. Rev. Saúde Pública.

[16] Folstein M.F., Folstein S.E., McHugh P.R. (1975). “Mini-Mental State”. A Practical Method for Grading the Cognitive State of Patients for the Clinician. J. Psychiatr. Res..

[17] Laks J., Baptista E.M.R., Contino A.L.B., de Paula E.O., Engelhardt E. (2007). Mini-Mental State Examination Norms in a Community-Dwelling Sample of Elderly with Low Schooling in Brazil. Cad. Saúde Pública.

[18] Bertolucci P.H., Brucki S.M., Campacci S.R., Juliano Y. (1994). The Mini-Mental State Examination in a General Population: Impact of Educational Status. Arq. Neuropsiquiatr..

[19] Schaakxs R., Comijs H.C., Lamers F., Beekman A.T.F., Penninx B.W.J.H. (2017). Age-Related Variability in the Presentation of Symptoms of Major Depressive Disorder. Psychol. Med..

[20] Wilder M.E., Kulie P., Jensen C., Levett P., Blanchard J., Dominguez L.W., Portela M., Srivastava A., Li Y., McCarthy M.L. (2021). The Impact of Social Determinants of Health on Medication Adherence: A Systematic Review and Meta-Analysis. J. Gen. Intern. Med..

[21] Comijs H.C., Nieuwesteeg J., Kok R., van Marwijk H.W., van der Mast R.C., Naarding P., Voshaar R.C.O., Verhaak P., de Waal M.W., Stek M.L. (2015). The Two-Year Course of Late-Life Depression; Results from the Netherlands Study of Depression in Older Persons. BMC Psychiatry.

[22] Vitorino L.M., Lopes J., Souza Santos G., Oliveira C., Helena J., Sousa L. (2023). Prevalence of Polypharmacy of Older People in a Large Brazilian Urban Center and its Associated Factors. Int. J. Environ. Res. Public Health.

[23] Livingston G., Huntley J., Liu K.Y., Costafreda S.G., Selbæk G., Alladi S., Ames D., Banerjee S., Burns A., Brayne C. (2024). Dementia Prevention, Intervention, and Care: 2024 Report of the Lancet Standing Commission. Lancet.

[24] Yu X., Qian Y., Zhang Y., Chen Y., Wang M. (2024). Association between Polypharmacy and Cognitive Impairment in Older Adults: A Systematic Review and Meta-Analysis. Geriatr. Nurs..

[25] Xue L., Boudreau R.M., Donohue J.M., Zgibor J.C., Marcum Z.A., Costacou T., Newman A.B., Waters T.M., Strotmeyer E.S. (2021). Persistent Polypharmacy and Fall Injury Risk: The Health, Aging and Body Composition Study. BMC Geriatr..

[26] Palapinyo S., Methaneethorn J., Leelakanok N. (2021). Association between Polypharmacy and Depression: A Systematic Review and Meta-Analysis. J. Pharm. Pract. Res..

[27] Wiersema C., Oude Voshaar R.C., van den Brink R.H.S., Wouters H., Verhaak P., Comijs H.C., Jeuring H.W. (2022). Determinants and Consequences of Polypharmacy in Patients with a Depressive Disorder in Later Life. Acta Psychiatr. Scand..

[28] World Health Organization (2023). WHO Launches Commission to Foster Social Connection. https://www.who.int/news/item/15-11-2023-who-launches-commission-to-foster-social-connection.

[29] Maese E. (2023). Almost a Quarter of the World Feels Lonely. https://news.gallup.com/opinion/gallup/512618/almost-quarter-world-feels-lonely.aspx.

[30] Beekman A.T.F., Geerlings S.W., Deeg D.J.H., Smit J.H., Schoevers R.S., de Beurs E., Braam A.W., Penninx B.W.J.H., van Tilburg W. (2002). The Natural History of Late-Life Depression: A 6-year prospective study in the community. Arch. Gen. Psychiatry.

[31] Chui H., Gerstorf D., Hoppmann C.A., Luszcz M.A. (2015). Trajectories of Depressive Symptoms in Old Age: Integrating Age-, Pathology-, and Mortality-Related Changes. Psychol. Aging G.

[32] Zhao M., Chen Z., Xu T., Ping F., Tian F. (2023). Global Prevalence of Polypharmacy and Potentially Inappropriate Medication in Older Patients with Dementia: A Systematic Review and Meta-Analysis. Front. Pharmacol..

[33] Scheel J., Luttenberger K., Graessel E., Kratzer A., Donath C. (2022). Predictors of Falls and Hospital Admissions in People with Cognitive Impairment in Day-Care: Role of Multimorbidity, Polypharmacy, and Potentially Inappropriate Medication. BMC Geriatr..

[34] Ie K., Chou E., Boyce R.D., Albert S.M. (2021). Fall Risk-Increasing Drugs, Polypharmacy, and Falls among Low-Income Community-Dwelling Older Adults. Innov. Aging.

[35] Leelakanok N., D’Cunha R.R. (2018). Association between Polypharmacy and Dementia—A Systematic Review and Metaanalysis. Aging Ment. Health.

[36] de Godoi Rezende Costa Molino C., Rübel L., Mantegazza N., Bischoff-Ferrari H.A., Freystaetter G. (2023). Association of Polypharmacy with Cognitive Impairment in Older Trauma Patients: A Cross-Sectional Study. Eur. J. Hosp. Pharm..

[37] Lovett R., Bonham M., Benavente J.Y., Hosseinian Z., Byrne G.J., Diaz M.V., Bass M., Yao L., Adin-Cristian A., Batio S. (2023). Primary Care Detection of Cognitive Impairment Leveraging Health and Consumer Technologies in Underserved US Communities: Protocol for a Pragmatic Randomised Controlled Trial of the MyCog Paradigm. BMJ Open.

[38] Pettigrew C., Soldan A. (2019). Defining Cognitive Reserve and Implications for Cognitive Aging. Curr. Neurol. Neurosci. Rep..

[39] Zhang X., Jiao J., Guo N., Bo H., Xu T., Wu X. (2021). Association of Polypharmacy with Falls among Older Chinese Inpatients: A Nationwide Cohort Study. Geriatr. Gerontol. Int..

[40] Watanabe K., Umegaki H., Sugimoto T., Fujisawa C., Komiya H., Nagae M., Yamada Y., Kuzuya M., Sakurai T. (2021). Associations between Polypharmacy and Gait Speed according to Cognitive Impairment Status: Cross-Sectional Study in a Japanese Memory Clinic. J. Alzheimer’s Dis..

[41] Adam C.E., Fitzpatrick A.L., Leary C.S., Hajat A., Ilango S.D., Park C., Phelan E.A., Semmens E.O. (2023). Change in Gait Speed and Fall Risk among Community-Dwelling Older Adults with and without Mild Cognitive Impairment: A Retrospective Cohort Analysis. BMC Geriatr..

[42] Hoel R.W., Giddings Connolly R.M., Takahashi P.Y. (2021). Polypharmacy Management in Older Patients. Mayo Clin. Proc..

[43] Sousa L., Tomás J., Severino S., Valido S., Santos M.J., José H. (2024). Prevention of Falls in the Elderly: A Challenge in Patient Safety. Salud Sci. Technol..

[44] Bai W., Chen P., Cai H., Zhang Q., Su Z., Cheung T., Jackson T., Sha S., Xiang Y.-T. (2022). Worldwide Prevalence of Mild Cognitive Impairment among Community Dwellers Aged 50 Years and Older: A Meta-Analysis and Systematic Review of Epidemiology Studies. Age Ageing.

[45] World Health Organization (2025). WHOQOL: Measuring Quality of Life. https://www.who.int/tools/whoqol.

[46] Steffens D.C., Manning K.J., Wu R., Grady J.J. (2023). Effects of Longitudinal Changes in Neuroticism and Stress on Cognitive Decline. Am. J. Geriatr. Psychiatry.

[47] Zhu Y., Li C., Xie W., Zhong B., Wu Y., Blumenthal J.A. (2021). Trajectories of Depressive Symptoms and Subsequent Cognitive Decline in Older Adults: A Pooled Analysis of Two Longitudinal Cohorts. Age Ageing.

[48] Diniz B.S., Butters M.A., Albert S.M., Dew M.A., Reynolds C.F. (2013). Late-life depression and risk of vascular dementia and Alzheimer’s disease: Systematic review and meta-analysis of community-based cohort studies. Br. J. Psychiatry.

[49] Turner A.D., James B.D., Capuano A.W., Aggarwal N.T., Barnes L.L. (2017). Perceived Stress and Cognitive Decline in Different Cognitive Domains in a Cohort of Older African Americans. Am. J. Geriatr. Psychiatry.

[50] Power M., Quinn K., Schmidt S. (2005). Development of the WHOQOL-Old Module. Qual. Life Res..

[51] World Health Organization (2022). World Mental Health Report: Transforming Mental Health for All. https://www.who.int/publications/i/item/9789240049338.

[52] Park S.-H., Kwak M.-J. (2020). Performance of the Geriatric Depression Scale-15 with Older Adults Aged over 65 Years: An Updated Review 2000–2019. Clin. Gerontol..

[53] de Abrantes G.G., Souza G.G., Cunha N.M., da Rocha H.N.B., Silva A.O., Vasconcelos S.C. (2019). Depressive Symptoms in Older Adults in Basic Health Care. Rev. Bras. Geriatr. Gerontol..

[54] Uomoto K.E. (2023). Increasing Identification and Follow-up of Older Adult Depression in Primary Care. J. Prim. Care Community Health.

